# Responding to human trafficking among refugees: prevalence and test accuracy of a modified version of the adult human trafficking screening tool

**DOI:** 10.1186/s12889-024-18997-7

**Published:** 2024-06-24

**Authors:** Estella Alejandra Tambini Stollwerck, Ivo Rollmann, Hans-Christoph Friederich, Christoph Nikendei

**Affiliations:** Department for General Internal Medicine and Psychosomatics, Heidelberg University Hospital, Heidelberg, Germany

**Keywords:** Human trafficking, Modern slavery, Exploitation, Refugees, Screening tool, Identification

## Abstract

**Background:**

Human trafficking is a human rights violation and urgent public health challenge. It involves the exploitation of a person by means of force, intimidation or deceit and causes severe health risks. Though it occurs all over the world, its true extent is still unknown. Refugees are especially vulnerable to human trafficking due to language barriers and difficult living conditions. Therefore, the purpose of this study was to estimate the prevalence and design a screening tool to identify survivors of all forms of human trafficking among refugees in a German state registration and reception centre.

**Methods:**

In cooperation with the local authorities and the Ministry of Justice and for Migration Baden-Württemberg, we interviewed newly arrived refugees at an initial reception centre in Southern Germany to assess the prevalence of human trafficking. We used both a combination of the Adult Human Trafficking Screening Tool and a publication by Mumma et al. to assess all forms of human trafficking.

**Results:**

In total, 13 of the 176 refugees had experienced trafficking, which corresponded to a prevalence of 7.3% (95%-CI = [3.5%, 11.3%]). Across all languages the questionnaire had a sensitivity of 76.9% and a specificity of 84.0% at a recommended cut-off of six positive responses. The recommended cut-off differed slightly for the Arabic, Farsi, Turkish, and English version. In an exploratory descriptive analysis on subregions, refugees from West Africa had a substantially higher prevalence (33.3%, 8 out of 24) for human trafficking within our sample, especially women. However, when we excluded this region from our analysis, we found no significant gender difference for the rest of the sample.

**Conclusions:**

The high prevalence of trafficking in most regions, regardless of gender, suggests that more effort is needed to identify and protect all trafficked persons. The designed screening tool seems to be a promising tool to detect an especially vulnerable group of refugees and provides assistance in identifying survivors of human trafficking.

**Supplementary Information:**

The online version contains supplementary material available at 10.1186/s12889-024-18997-7.

## Introduction

Human trafficking involves the recruitment, movement or harbouring of persons by deception, the use of threats, coercion or a position of vulnerability for the purpose of exploitation [1]. Different forms of exploitation are forced labour, sexual exploitation, slavery or servitude, forced criminal activity as well as the removal of organs. Available data suggests that the majority of trafficked men are forced into labour exploitation, while women are mainly forced into domestic work, sexual exploitation and forced marriage [2, 3]. Until today, the media primarily portrays female survivors in need to be rescued from sex trafficking, oversimplifying the complexity of trafficking situations, neglecting male and LGBTQ victims [4, 5]. Human trafficking is a massive human rights violation of global proportions that creates a highly prevalent public health burden [3]. Physical injuries, illness and psychological morbidity result out of unfair employment terms, bad working conditions and experiences of violence. It is largely undetected, and it is still unknown how many people are truly trafficked today [6]. For a rough estimate, according to the European Commission, 14,000 trafficking survivors were registered in the European Union between 2017 and 2018 [7].

Migrants account for a considerably large share of detected victims of human trafficking and are especially vulnerable to promises about jobs, money, and security [2, 6, 8]. Because of a lack of knowledge about the local culture and regulations, the distance to relatives and friends, language barriers and socioeconomic hardship, they are more likely to get lured into trafficking [9]. For example, the West African subregion is the largest non-European region of origin to contribute to trafficking flows into Western and Southern Europe [6, 10]. Generally, a screening process to identify human trafficking among migrants of any country is an important step towards an improved victim identification, with a subsequent referral to support systems to accompany them during the asylum procedure and offer social and psychological counselling being essential.

There are various screening tools of different lengths to identify human trafficking. A review by Macy et al. [11] demonstrated that most of these instruments were developed by practice-oriented and non-governmental organizations in the U.S. and were therefore best suited for this particular population. Some were developed for specialized settings such as healthcare providers as they are often the only ones to talk to trafficked persons outside their trafficking situation [12, 13]. Recent reviews not only criticized the absence of a reliable gold standard, but also found that only few screening tools had been thoroughly evaluated [11, 14]. Two of these tools were the Adult Human Trafficking Screening Tool (AHTST) by Macias Konstantopoulos & Owens [15] and the screening for emergency settings by Mumma et al. [16]. The AHTST was designed to identify people currently or at risk of being trafficked. It provides a guideline to recognise the signs and red flags presented by victims of human trafficking in health care and social work based on self-disclosure. The trauma-informed screening tool contains eight questions about different trafficking situations. A positive result was defined as answering at least once with “yes” to any screening question. The original version, however, was not validated nor was its sensitivity and specificity published [15]. The AHTST is suitable for the application in different domains and has been used in different contexts [17]. For example, the tool succeeded in detecting three trafficked women out of 145 patients in two clinics in Oklahoma [13]. However, to our knowledge, the AHTST has solely been used in the United States of America and thus could be biased due to a Western, industrialised, wealthy, and democratic study population.

In a different approach, Mumma et al. [16] conducted a study in order to evaluate the feasibility of a screening instrument in the emergency department. Answering “yes” to any of the screening questions was defined as a positive survey screen. Among 143 participants, 39 were initially screened positive, including ten that were ultimately identified as victims of sex trafficking. All ten “true positive” cases answered one question about abuse positively which, according to the authors, could suffice for extremely quick assessments. The sensitivity of the overall screening tool with 14 questions was 100% [CI 70-100%], specificity was 78% (CI [70–85%]). However, its focus on sex trafficking limits the overall screening to just one form of trafficking in persons.

Thus, the present study has two goals: (1) Estimating the prevalence of human trafficking in a German state registration and reception centre and (2) to assess whether the AHTST could also be used in a European sample.

## Methods

### Study design and ethical considerations

This study is a prospective cross-sectional study that sought to conduct a survey on the topic of human trafficking among refugees in a state registration and reception centre. It intended to assess the prevalence rate as well as sensitivity and specificity of the respective instrument. The study was approved by the local ethics committee (Ethics Committee, Medical Faculty of Heidelberg University, S249/2021; 05.05.2021) and was in line with the Declaration of Helsinki.

### Sample size and power calculation

To the best of our knowledge, no systematic study about the prevalence of trafficking among refugees within a state registration and reception centre in Germany exists currently. Reports from the International Organization for Migration estimated in 2016 that about 80% of all Nigerian women and girls arriving by sea in Italy or other countries of the European Union were likely to be targeted for sexual exploitation [18]. In 2017, the IOM reported that 37% of all interviewed migrants who had taken Mediterranean routes to Europe had personal experience indicating human trafficking [10]. However, as there are many male refugees among the refugees in Germany who did not necessarily take the Mediterranean route, we estimated the percentage to be lower. Therefore, we estimated the prevalence for trafficking experiences among refugees from any world region conservatively to be around 10%. Furthermore, the results of the AHTST and the single question by Mumma et al. [16] indicate that trafficking survivors differ significantly in their responses from non-trafficked persons. Therefore, we expected our questionnaire to have a sensitivity of 0.90 and followed the recommendations of Bujang et al. [19] for the minimum required sample size: They calculated that the minimum required sample size to achieve a significance level of α = 0.05 and a power β = 0.80 is 120 people, with at least 12 of them being positive.

### Participants

Our target group were all refugees in the state registration and reception centre who fulfilled our inclusion criteria. Inclusion criteria were assessed at first contact and included an age of 18 or older, literacy, and the ability to understand one of the following languages: Arabic, German, English, French, Farsi, Georgian, Kurmanji, Hausa, Pashto, Serbian, Somali, Turkish, and, due to the high number of refugees fleeing from the war in Ukraine after March 2022, also Russian and Ukrainian. Any person not fulfilling any of these criteria was excluded from the study. A total of 176 people took part in the study. Within our three gender categories, 35 (19.9%) identified as being female, 140 (79.5%) as being male and one (0.6%) person as being diverse. The average age was 30.9 years (SD = 8.2 years). 84 (47.7%) of the participants had children. As can be seen in Table 1, most participants came from West Asia and Central Asia. Accordingly, Arabic, Turkish and Farsi were the most commonly spoken languages and Islam the most common religion.

**Table 1:** Regions of origin, languages and religion of all participants (*N* = 176)

**Region of Origin**	**Percentage**		**N**
West Asia	38.1		67
Central Asia	17.6		31
East Europe	16.5		29
West Africa	13.6		24
North Africa	11.4		20
East Africa	1.1		2
Bahamas	0.6		1
Unknown	1.1		2
**Language**	**Percentage**		**N**
Arabic	25.0		44
Turkish	19.3		34
Farsi	16.5		29
English	14.2		25
Georgian	10.2		18
French	7.4		13
Russian	2.3		4
German	2.3		4
Serbian	1.7		3
Pashtu	0.6		1
Ukrainian	0.6		1
**Religion**	**Percentage**		**N**
Islam	66.5		117
Christianity	25.6		45
Atheism	3.9		7
Alevis	1.1		2
Hinduism	0.6		1
Other	2.3		4

*Note* The allocation of individual countries to regions is found at 10.11588/data/DUNN8C. The order is based on the percentages

### Setting

The Patrick Henry Village Heidelberg (PHV) is where asylum-seekers are registered for the first time in the state of Baden-Württemberg. It usually accommodates between 1200 and 2400 newly arrived asylum-seekers. During their stay at PHV, state employees verify their identity, register their personal data and carry out a medical examination for communicable diseases as part of the asylum procedure. After around five weeks, asylum-seekers are redistributed to communal accommodations.

### Development of the screening tool

In our study, we used a combination of the eight questions of the AHTST [15] and the single question about abuse which was developed by Mumma et al. [16]. The AHTST applies to different forms of human trafficking while the screening questions by Mumma et al. [16] originally aimed at the identification of victims of sex trafficking in the emergency department but was so accurate in the original publication that we included it in our questionnaire (see Appendix A). We considered both valuable instruments for all forms of exploitation that were trauma-informed and culturally sensitive, which we assumed to be especially important for our sample of refugees. Hereby, we defined cultural sensitivity as an ability to meet the participants’ social and cultural needs, even though they come from different backgrounds with diverse believes or values. For each of the presented items, there were four answer categories: “Yes”, “No”, “Don’t know”, and “I Decline to answer”. Other than in the U.S. population, for which the original version of the AHTST was created, we expected a threshold of one positive answer as too low for our context, as most asylum-seekers experience similar forms of violence outside of a trafficking situation [20]. Certified translators translated the English version into eleven other languages (Arabic, Farsi, French, German, Georgian, Hausa, Kurmanji, Pashto, Serbian, Somali, Turkish), and when the numbers of Ukrainian refugees increased in March 2022, we also provided a Russian and Ukrainian version of our study. All translated versions of the questionnaire are available online at: 10.11588/data/DUNN8C.

### Data collection

The study took place during the Covid-19 pandemic in late 2021 until summer 2022. During the first four months of the study, all participants had to remain under quarantine for ten to twelve days due to pandemic requirements. During their fifth to eighth day of isolation, when they were already tested negative but were only allowed to step out a few meters outside, we interviewed the participants in front of their accommodation. From March 2022 onwards, the quarantine for refugees wasn’t obligatory anymore, so they were free to go outside anytime. The screening was restricted by specific precautions, such as wearing a mask or keeping a distance of two metres between participants and two female researchers. The researchers approached all potential participants in front of their accommodations one at a time. There, the following data was collected:

### Demographic information and screening tool

In this phase, participants used a tablet that the researchers provided. They completed several demographic questions concerning their age, gender, country of origin, religion, and parenthood. Next, participants filled out the Adult Human Trafficking Screening Tool followed by the central question found in Mumma et al. [16], as described earlier.

### Verification of human trafficking

Independent from their answers provided in the screening tool, every participant answered five additional questions about indicators for human trafficking. In this short semi-structured interview, we cross-checked whether a participant had experienced trafficking according to the questions below about working hours, imprisonment, control over payment, experiences of violence and debt. The researcher posed the following questions with the support of a multilingual neural machine translation within five to ten minutes and evaluated the narratives individually.

- Have you ever been in a situation where you had to work for long hours every day without any day off?
- Have you ever been in a situation where you worked in a house that you were not allowed to leave?
- Have you ever worked and someone else was in control of your income?
- Have you ever been in a situation where you or someone you worked with was beaten and made feel pain for working slowly or trying to leave?
- Have you ever been forced to work because you felt you were bonded by debt?

Participants that described trafficking experiences were invited to present themselves at a psychosocial walk-in clinic [21] or at the local counselling service. If the trafficking survivors gave their consent, the researchers reached out to the local specialised counselling centre for trafficked people, social workers within the state registration and reception centre, and the coordinator for accommodation of asylum-seekers of vulnerable groups.

### Data analysis

All of our analyses are available online at: 10.11588/data/DUNN8C.

All calculations were performed with Microsoft Excel 2019. All percentages were calculated using proportions. Confidence Intervals were calculated using gaussian standard normal quantiles, as our sample size surpassed 30 in each cases. We first added up how many questions the participants answered with “Yes”. Then, for each possible cut-off of one to nine “Yes” answered questions, we calculated how many True Positive, False Positive, True Negative, and False Negative results the questionnaire yielded. With this, we calculated the sensitivity, specificity, positive predictive value, negative predictive value, correct classification rate, and positive likelihood ratio of the questionnaire. The choice of the final cut-off was based on several assumptions: Since the group of people who did not experience human trafficking was expected to be larger, we aimed at a cut-off that would generate as few false positives as possible. At the same time, the cut-off, if it were to indicate a positive result, should be relevant and clearly indicate human trafficking. We therefore also chose the cut-off with the highest possible relevance and positive likelihood ratio. These assumptions were made as we assume that our screening tool will mostly be used in state registration and reception centres. The aim here was to select a cut-off that would eliminate as many false positives as possible in order to burden organisational processes as little as possible with screening interviews. At the same time, we are aware that other cut-offs would be more suitable for other research questions. Therefore, we have made our complete analysis publicly available online at the following link: 10.11588/data/DUNN8C. We carried out this sensitivity analysis for the overall sample across all languages and separately for all languages used by at least 30 participants. However, as this study was primarily intended to record the prevalence of human trafficking, the sample sizes for the individual languages were very small and therefore underpowered. As described in the introduction, the West African subregion is the non-European region of origin with the highest contribution to trafficking flows into Western and Southern Europe [6]. We therefore decided to conduct an additional exploratory analysis comparing West African participants with the rest of our sample. Because only 24 of 176 participants were from the West African region, this analysis resulted in a particularly uneven sample size. Since this uneven sample size did not provide enough power for significance testing, the comparison was descriptive.

## Results

### Participation rates

During the survey period, 1426 people lived in the accommodations that were visited by the research assistants, including both adults and their children. 343 of them were asked to participate in the study. Of the 343 people approached, 45 did not meet our inclusion criteria. Of these, 35 did not speak any of the 14 languages, 8 were illiterate, and 2 were under 18 years old. Of the remaining 298 individuals, 120 refused to participate, resulting in a participation rate of 59.7%. The majority declined to participate without stating reasons. Two participants dropped out of the survey, leaving a total of 176 participants in the study.

### Prevalence of human trafficking

Of the 176 participants in our study, we identified 13 who experienced human trafficking according to their own information in a short semi-structured interview after the screening assessment. This corresponds to a proportion of 7.3% (95%-CI = [3.5%, 11.3%]). Among these 13 people, five participants were exploited sexually (two men, three women), eight had experienced labour exploitation (five men, three women), and one female participant additionally reported she was exploited within a forced marriage. In other words, within our sample of 35 women and 140 men, the percentage having experienced trafficking as a woman was 17.1% (6 out of 35, 95%-CI = [4.7%, 29.6%]). As a man, the percentage was 5.0% (7 out of 140, 95%-CI = [1.4%, 8.6%]).

### Calculations of cut-offs

**Fig. 1:**
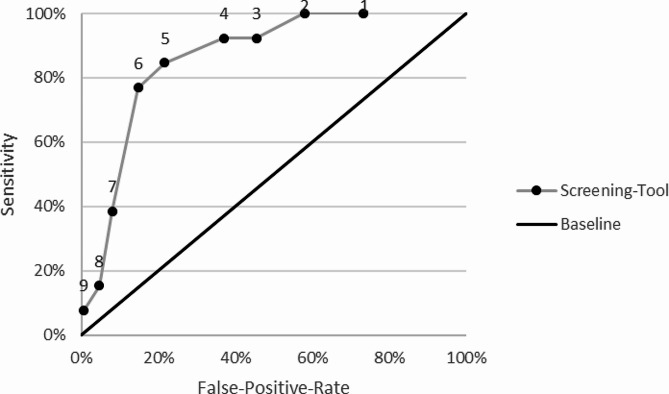
Receiver-Operating-Curve of the questionnaire for the overall sample. *Note* Numbers above points indicate possible cut-off for our screening tool. Sensitivity: Sensitivity our screening tool would have with the respective cut-off. False-Positive-Rate: Percentage of false positives one would expect for respective cut-off. Baseline is included to compare performance of the test, with a test which has sensitivity and specificity of 0. Results are based on a sample size of *N* = 176

The results of our analysis are displayed in Table 2; Fig. 1. On average, we decided on a cut-off value of 6 questions answered with “Yes” for a positive screening result. This decision was made because this cut-off was most in line with our assumptions. With a sensitivity of 76.7% and a specificity of 84.0%, this cut-off captures most survivors of human trafficking and generates few false positives. It also has the second highest positive likelihood ratio and the second highest relevance. In other words, a positive result with this cut-off strongly indicates human trafficking. In our sample, ten out of 13 trafficked people screen positive when applying a cut-off of six questions answered with “yes”. However, we did observe that the optimal cut-off values differ between languages. According to the same assumptions, a cut-off of 5 is optimal in Arabic and English, while in contrast, a cut-off of 7 is optimal in Farsi and Turkish. We further observed that a cut-off of only 1 question answered with “yes”, as recommended in the American version of the AHTST [15], only correctly classified about 9 to 52% of the current sample.

**Table 2:** Analysis of possible cut-offs for the screening tool, overall and different languages

**Overall estimation**						
**Cut-Off**	**Sensitivity**	**Specificity**	**PPV**	**NPV**	**CCR**	**PLQ**
1	100.0%	20.9%	9.2%	100.0%	26.7%	1.3
2	100.0%	37.4%	11.3%	100.0%	42.0%	1.6
3	92.3%	50.9%	13.0%	98.8%	54.0%	1.9
4	92.3%	60.1%	15.6%	99.0%	62.5%	2.3
5	84.6%	76.7%	22.4%	98.4%	77.3%	3.6
**6**	**76.9%**	**84.0%**	**27.8%**	**97.9%**	**83.5%**	**4.8**
7	38.5%	91.4%	26.3%	94.9%	87.5%	4.5
8	15.4%	95.1%	20.0%	93.4%	89.2%	3.1
9	7.7%	99.4%	50.0%	93.1%	92.6%	12.5
**Arabic**						
**Cut-Off**	**Sensitivity**	**Specificity**	**PPV**	**NPV**	**CCR**	**PLQ**
1	100.0%	7.1%	4.9%	100.0%	11.4%	1.1
2	100.0%	35.7%	6.9%	100.0%	38.6%	1.6
3	100.0%	45.2%	8.0%	100.0%	47.7%	1.8
4	100.0%	57.1%	10.0%	100.0%	59.1%	2.3
**5**	**100.0%**	**78.6%**	**18.2%**	**100.0%**	**79.5%**	**4.7**
6	50.0%	85.7%	14.3%	97.3%	84.1%	3.5
7	0.0%	90.5%	0.0%	95.0%	86.4%	0.0
8	0.0%	95.2%	0.0%	95.2%	90.9%	0.0
9	0.0%	100.0%	NA	95.5%	95.5%	NA
**Turkish**						
**Cut-Off**	**Sensitivity**	**Specificity**	**PPV**	**NPV**	**CCR**	**PLQ**
1	100.0%	6.1%	3.1%	100.0%	8.8%	1.1
2	100.0%	18.2%	3.6%	100.0%	20.6%	1.2
3	100.0%	30.3%	4.2%	100.0%	32.4%	1.4
4	100.0%	45.5%	5.3%	100.0%	47.1%	1.8
5	100.0%	66.7%	8.3%	100.0%	67.6%	3.0
6	100.0%	81.8%	14.3%	100.0%	82.4%	5.5
**7**	**100.0%**	**90.9%**	**25.0%**	**100.0%**	**91.2%**	**11.0**
8	0.0%	93.9%	0.0%	96.9%	91.2%	0.0
9	0.0%	100.0%	NA	97.1%	97.1%	NA
**Farsi**						
**Cut-Off**	**Sensitivity**	**Specificity**	**PPV**	**NPV**	**CCR**	**PLQ**
1	100.0%	50.0%	6.7%	100.0%	51.7%	2.0
2	100.0%	60.7%	8.3%	100.0%	62.1%	2.5
3	100.0%	75.0%	12.5%	100.0%	75.9%	4.0
4	100.0%	78.6%	14.3%	100.0%	79.3%	4.7
5	100.0%	85.7%	20.0%	100.0%	86.2%	7.0
6	100.0%	85.7%	20.0%	100.0%	86.2%	7.0
**7**	**100.0%**	**89.3%**	**25.0%**	**100.0%**	**89.7%**	**9.3**
8	0.0%	96.4%	0.0%	96.4%	93.1%	0.0
9	0.0%	100.0%	NA	96.6%	96.6%	NA
**English**						
**Cut-Off**	**Sensitivity**	**Specificity**	**PPV**	**NPV**	**CCR**	**PLQ**
1	100.0%	31.6%	31.6%	100.0%	48.0%	1.5
2	100.0%	42.1%	35.3%	100.0%	56.0%	1.7
3	83.3%	63.2%	41.7%	92.3%	68.0%	2.3
4	83.3%	68.4%	45.5%	92.9%	72.0%	2.6
**5**	**66.7%**	**84.2%**	**57.1%**	**88.9%**	**80.0%**	**4.2**
6	66.7%	84.2%	57.1%	88.9%	80.0%	4.2
7	16.7%	94.7%	50.0%	78.3%	76.0%	3.2
8	16.7%	94.7%	50.0%	78.3%	76.0%	3.2
9	16.7%	94.7%	50.0%	78.3%	76.0%	3.2

*Note* Highlighted value represent best value according to our assumptions. *PPV* positive predictive value, *NPV* negative predictive value, *CCR* correct classification rate, *PLQ* positive Likelihood-Quotient, *NA* not estimateable as no person in sample achieved that cut-off

### Subsample analysis of west African participants

The results of our explorative descriptive analysis can be seen in Table 3. The comparison between West African refugees and participants from other regions revealed a difference in the frequency of being trafficked. 8 of 24 refugees from West Africa experienced trafficking, compared to only 5 in 151 refugees from other regions. In other words, the proportion of refugees from West Africa which experienced trafficking was 10 times higher. In an explorative analysis of gender differences, we found that women from West Africa (5 out of 9) were 14 times more likely to be trafficked than women from other regions (1 out of 26). Men from West Africa (3 out of 15) were six times more likely to be trafficked than men from other regions (4 out of 125). Furthermore, in the sample excluding West Africa, we found no difference between men and women in the prevalence of being trafficked. A post hoc confidence interval for the difference in the proportion of trafficked women and men in our sample excluding west Africa was not significant, 95%-CI = [-0.074, 0.087].

**Table 3:** Comparison of West African refugees with the rest of the researched sample

	West African refugees		Rest of sample	
	N	Percentage	N	Percentage
Total	24	100	152	100
Female	9	37.5	26	17.1
Male	15	62.5	125	82.2
Diverse	0	0.0	1	0.7
With Children	11	45.8	73	48.0
HTS	8 of 24	33.3	5 of 152	3.3
Female HTS	5 of 9	55.6	1 of 26	3.8
Male HTS	3 of 15	20.0	4 of 125	3.2

*NoteHTS* Human Trafficking Survivor

## Discussion

To our knowledge, this study is the first to systematically estimate the prevalence of human trafficking in a German state registration and reception centre among refugees. For this population, we estimated the prevalence of human trafficking to be 7.3% (95%-CI = [3.5%, 11.3%]). Overall, refugee women and men were equally affected by human trafficking, except for West Africa. Furthermore, our results suggest that the AHTST, extended with the question by Mumma et al. (15; 16), seems to be suitable to be used as a screening tool for all forms of human trafficking. Nevertheless, the cut-off should be adjusted. With a cut-off of one question answered with “yes”, the screening would have only correctly classified 9 to 52%, depending on the language. Across all languages, a cut-off of 6 questions answered with “yes” achieved a sensitivity of 76.9% and a specificity of 84.0%. Yet, in our sample, the cut-off value for our assumptions would be between 5 and 7 questions answered with “yes”, depending on the language. We assumed the context that the screening will be used in state registration and reception centres, so future researchers are free to use other cut-off values depending on their research question. All results can be found online at: 10.11588/data/DUNN8C. We further identified survivors of trafficking among vulnerable subgroups, such as West African refugees, as well as trafficked men, who are rarely the focus of research on trafficking [22]. Therefore, we deem the screening tool useful as a means to quickly and easily screen all forms of human trafficking for all genders in fourteen languages.

The exploratory findings may provide new insight to gender differences among survivors of trafficking, as we found male refugees to be as vulnerable as women in all subregions but West Africa within our sample. Within our sample, women from West African countries were being trafficked more frequently, which is consistent with former reports on a high prevalence in this part of the world [10, 18]. Overall, we collected more data from male participants due to the fact that we encountered more men in the state registration and reception centre. Furthermore, the fact that we recommend a positive screening result only after 6 questions have been answered with “yes”, compared to 1 [15, 16], indicates that a high percentage of refugees had experienced similar forms of violence in contexts other than a trafficking situation. The rate of true positive-screens was high, particularly given the assumption that survivors of trafficking could be hindered by fear, shame or distrust [23], and the fact that they were still waiting for a response to their asylum application.

Our study aimed to estimate the prevalence of human trafficking among refugees and assess whether a standardised screening in a state registration and reception centre for asylum-seekers with a high vulnerability to human trafficking is feasible. The participants were often curious, seldom reluctant, and those that had experienced trafficking were especially thankful to share their story. The screening tool is easy to assess, quick, and trauma-informed. It could therefore be used in various contexts, for all forms of exploitation and all genders. It therefore serves well as pre-selection to indicate which refugees are most likely to have been trafficked. Nevertheless, human trafficking is a complex crime that varies substantially from case to case. Therefore, it is not possible to base the final decision on whether someone has experienced trafficking on a screening tool alone. Migrants are exposed to extreme violence during their flight, including incarceration, pressure to earn money for a living or to pay smugglers (e.g. survival sex). Others may be sold into forced marriage within their home countries and may not get paid for endless work which also counts as Modern Slavery [24]. To call this human trafficking is a matter of interpretation, which is why we recommend conducting a longer conversation after the fact to clarify whether and how human trafficking has occurred.

### Limitations

This study has several limitations. First, as for all studies about human trafficking, no gold standard to identify survivors exists. It is possible that trafficked asylum-seekers were overlooked because they had false negative screens, or that they were hindered by language barriers due to the use of the translation machine. The generalisation of our results is limited by the fact that we could not survey all refugees because some did not meet the inclusion criteria. They either did not speak any of the 14 languages we provided or were illiterate. Furthermore, we did not check for language comprehension skills at the beginning of the screening, which might have influenced the results. Therefore, some of our results might be biased, as illiterate people, a likely vulnerable group for human trafficking, were left out in our sample. Furthermore, the study took place under Covid restrictions and only those who wanted to talk to us outside the accommodation were able to participate. The main statistical limitation of our study is that our primary aim was to estimate the prevalence of human trafficking. As a result, we did not ensure that all languages were used equally and some languages were only used once. Although we conducted a sensitivity analysis for the four most common languages and across all languages, these results should be verified in future studies. For the individual languages, the sample is too small, leading to insufficient power. On the other hand, it is not methodologically permissible to transfer results from one language to another, as can be seen in our results between Arabic and Turkish. While we believe a cut-off of 6 questions answered with “yes” to be a plausible estimation, future studies would need to validate our results. Furthermore, as we had no insight of the gender profile of the state registration and reception centre during the time of our study, it was not possible to ascertain that the gender profile in our sample corresponds to it. Moreover, as we had no data from people declining participation, it was not possible to assess whether this missing data was statistically ‘missing completely at random’ or ‘missing not at random’. It must therefore be assumed that our results are still subject to bias, which should be analysed in future studies. In line with this limitation is the fact that all our interviewers were female, while most participants were male. There might have been an interaction effect taking place. Future studies should thus conduct their study with male and female interviewers. The use of interpreters could address this problem and include illiterate people if the resources are available. Next, our exploratory analysis on the West African subgroup was mostly descriptive due to the uneven sample sizes. As a result, we did not have enough power for significance testing. Finally, this study focussed on adult survivors of human trafficking and is not intended for use with minors.

### Implications for future research

Future studies might focus on the subgroup of refugees from West Africa as, in our sample, trafficking was highly prevalent in this region. In our descriptive analysis, women from West African countries were almost fifteen times more likely to become trafficked than women from other regions. For all other regions, the prevalence for trafficking was almost equally high among men and women, which was highly surprising. The numbers for the entire sample were so heavily skewed by the West African subset that there originally appeared to be a gender gap for the total sample. This is a lesson learned: Even if the overall perception suggests that women are particularly affected by human trafficking, it is always worth taking a closer look. If future studies with larger samples show similar results, this proves that men from other subregions than West Africa are just as vulnerable to human trafficking as other genders.

## Conclusion

This paper proposes a new approach to the identification of trafficked people during the initial registration of asylum seekers, responding to a highly prevalent public health burden [3]. Applied on a broad scale, more asylum-seekers could receive support upon their arrival in receiving countries [7]. This is the first study to systematically document the prevalence of human trafficking among refugees in Germany with a practice-oriented screening tool. Therefore, our research assists in the detection of especially vulnerable people within state facilities.

### Electronic supplementary material

Below is the link to the electronic supplementary material.

Supplementary Material 1

## Data Availability

The datasets generated and/or analysed during the current study are available in the Heidelberg Open Research Data (heiDATA) repository, permanent link: 10.11588/data/DUNN8C.
