# Supplementary material for: Responding to human trafficking among refugees: prevalence and test accuracy of a modified version of the adult human trafficking screening tool
Source: BMC Public Health. 2024 Jun 24;24:1685. doi: 10.1186/s12889-024-18997-7 (PMC11197328; doi:10.1186/s12889-024-18997-7)
Supplement: Supplementary file 1 — Supplementary Material 1 [file 12889_2024_18997_MOESM1_ESM.docx]

**Appendix A: The combination of the two screening tools used in this study**

| **Adult Human Trafficking Screening Tool** (Macias Konstantopoulos & Owens, 2018) |
| --- |
| 1. Sometimes lies are used to trick people into accepting a job that doesn’t exist, and they get trapped in a job or situation they never wanted. |
| 1. Sometimes people make efforts to repay a person who provided them with transportation, a place to stay, money, or something else they needed. The person they owe money to may require them to do things if they have difficulty paying because of the debt. |
| 1. Sometimes people do unfair, unsafe, or even dangerous work or stay in dangerous situation because if they don't, someone might hurt them or someone they love. |
| 1. Sometimes people are not allowed to keep or hold on to their own identification or travel documents. |
| 1. Sometimes people work for someone or spend time with someone who does not let them contact their family, spend time with their friends, or go where they want when they want. |
| 1. Sometimes people live where they work or where the person in charge tells them to live, and they’re not allowed to live elsewhere. |
| 1. Sometimes people are told to lie about their situation, including the kind of work they do. |
| 1. Sometimes people are hurt or threatened, or threats are made to their family or loved ones, or they are forced to do things they do not want to do in order to make money for someone else or to pay off a debt to them. |
| *All questions were followed by the subsequent question and response categories:*  Have you ever experienced this, or are you in a situation where you think this could happen?  Yes – No – I Decline to Answer – Don’t Know |
| **Additional question created by Mumma et al. (2017):**  Were you [or anyone you work with] ever beaten, hit, yelled at, raped, threatened or made to feel physical pain for working slowly or for trying to leave?  Yes – No – I Decline to Answer – Don’t Know |
